# The intersection of TREX1, cGAS, STING and the DNA damage theory of aging

**DOI:** 10.3389/fragi.2025.1720372

**Published:** 2025-12-04

**Authors:** Kate M. Jones, Samuel D. Chauvin, Jonathan J. Miner

**Affiliations:** 1 Division of Rheumatology, Department of Medicine, University of Pennsylvania Perelman School of Medicine, Philadelphia, PA, United States; 2 RVCL Research Center, University of Pennsylvania Perelman School of Medicine, Philadelphia, PA, United States; 3 Department of Microbiology, University of Pennsylvania Perelman School of Medicine, Philadelphia, PA, United States; 4 Institute for Immunology and Immune Health, University of Pennsylvania Perelman School of Medicine, Philadelphia, PA, United States; 5 Colton Center for Autoimmunity, University of Pennsylvania Perelman School of Medicine, Philadelphia, PA, United States

**Keywords:** TREX1, retinal vasculopathy with cerebral leukoencephalopathy, RVCL, RVCL-S, Aicardi-Goutières syndrome, AGS, STING, aging

## Abstract

Genetic syndromes of immune dysregulation have opened a door toward understanding mechanisms linking inflammation, premature aging, and normal aging. Here, we discuss new insights into the relationship between DNA damage, premature senescence, and nucleic acid-sensing pathways that detect or regulate DNA damage. First, we review mechanisms by which the DNA exonuclease TREX1 negatively regulates the cytosolic DNA sensor cGAS and its downstream effector STING, and we propose a model of TREX1-mediated DNA damage and cellular senescence that implicates age-related, inducible TREX1 expression in the context of genetic disease and inflamm-aging. Our central thesis is that two TREX1-associated diseases—Aicardi-Goutières syndrome (AGS) and retinal vasculopathy with cerebral leukoencephalopathy (RVCL or RVCL-S), historically regarded as inflammatory conditions—can serve as models for research into mechanisms of premature aging.

## Introduction

One of the leading theories of aging posits that the progressive accumulation of DNA damage is the primary driver of the aging process (Niedernhofer et al., 2018; Schumacher et al., 2021). Throughout life, cells are continuously exposed to endogenous and environmental insults that induce DNA lesions. Although most damage is efficiently repaired, a small fraction persists or is misrepaired, leading to its gradual accumulation with age (Niedernhofer et al., 2018; Schumacher et al., 2021). The inexorable drive towards increasing levels of DNA damage results in many of the downstream consequences that characterize aging, such as cellular senescence and inflammation (Niedernhofer et al., 2018; Schumacher et al., 2021; Zhao et al., 2023). Therefore, it should not be surprising that many progeroid syndromes result from genomic instability as a consequence of DNA damage or impaired DNA repair. Loss of *WRN*, which encodes a DNA helicase, causes genomic instability and the premature aging phenotype of Werner syndrome (Oshima et al., 2017). Likewise, mutations in *LMNA* result in impaired nuclear membrane integrity and defective DNA repair, eventually leading to the early aging observed in Hutchinson-Gilford progeria syndrome (Gonzalo and Kreienkamp, 2015). Thus, understanding the role of DNA damage is essential to defining mechanisms driving the pathogenesis of progeroid syndromes as well as physiologic aging.

Chronic inflammation is a hallmark of aging (López-Otín et al., 2013; Franceschi et al., 2000). Inflammatory cytokines such as IL-6, IFN-α and TNF-α are up-regulated even in otherwise healthy older adults (Ferrucci and Fabbri, 2018). This inflammation is driven in part by cellular senescence, which can be triggered by cellular insults including DNA damage (Ferrucci and Fabbri, 2018). Likewise, inflammation itself can contribute to the induction of the senescence-associated secretory phenotype (SASP), which accelerates the aging process (Wang et al., 2024). Indeed, senolytic and senomorphic drugs, which either kill senescent cells or suppress the SASP, can extend the lifespan and healthspan of mice (Chaib et al., 2022; Zhang et al., 2023). For example, the mTOR inhibitor rapamycin is a senomorphic drug that reduces NF-κB-dependent cytokines and extends the lifespan of mice by ∼10%, supporting a link between mTOR, metabolism, and the inflammation of aging (Harrison et al., 2009; Lamming et al., 2012; Dan et al., 2008). In further confirmation on the role of inflammation in aging, a recently identified progeroid syndrome is caused by absence of caspase 5 associated with elevated levels of pro-inflammatory cytokines (Hisama et al., 2024). Direct blockade of cytokine signaling has also been proposed as an intervention in aging. Recent work by Widjaja and colleagues revealed that genetic deletion of IL-11, as well as anti-IL-11 antibody therapy, increases the lifespan of mice by more than 20% (Widjaja et al., 2024). Hence, understanding mechanisms of age-related inflammation is crucial for developing interventions for the treatment of aging-related disease.

Three prime repair exonuclease 1 (TREX1) sits at the intersection of many of the most important pathways involved in aging, including DNA damage and systemic inflammation. TREX1, the most abundant and highly potent mammalian 3′-5′ DNA exonuclease, degrades cytosolic DNA to prevent activation of the cGAS-STING pathway (Wang et al., 2022; Gall et al., 2012; Mazur and Perrino, 2001; Simpson et al., 2020). N-terminal mutations in TREX1 disrupt its exonuclease domain and limit its ability to degrade cytosolic DNA (Wang et al., 2022; de Silva et al., 2007). Thus, N-terminal TREX1 mutations result in hyperactivation of the cGAS-STING pathway, causing inflammatory diseases such as Aicardi-Goutières syndrome (AGS) and Familial chilblain lupus (FCL) (Wang et al., 2022; Rice et al., 2007; Liu and Ying, 2023). Inflammation mediated by constitutive cGAS-STING activity, which occurs in the context of TREX1 deletion, ultimately results in DNA damage and cellular senescence (Gao et al., 2015; Giordano et al., 2022; Du et al., 2023). Notably, aberrant cGAS-STING activation also mediates inflammation in progeroid syndromes such as Hutchinson-Gilford progeria syndrome (Paul et al., 2021; Kreienkamp et al., 2018). Therefore, N-terminal TREX1 mutations provide a useful platform for exploring the role of inflammation in the aging process. This concept is further supported by recent discoveries indicating that cytokines cause induction of TREX1, and that up-regulation of TREX1 – and in particular the up-regulation of a nuclear-localized form of TREX1 – occurs concomitantly with DNA damage (Chauvin et al., 2024; Serra et al., 2011). Thus, studying TREX1 allows for mechanistic exploration of cytokine drivers of DNA damage and aging.

C-terminal TREX1 mutations cause genomic instability and cellular senescence, but through a distinct molecular mechanism not associated with systemic inflammation. C-terminal mutations in TREX1 disrupt its ER-anchoring transmembrane domain, leading to mislocalization of an active exonuclease domain within the nucleus, resulting in chromosomal injury, cell death, and cellular senescence without aberrant activation of the cGAS-STING pathway (Chauvin et al., 2024). These autosomal dominant C-terminal TREX1 mutations cause a devastating adult-onset disease known as retinal vasculopathy with cerebral leukoencephalopathy (RVCL or RVCL-S), a DNA damage syndrome characterized by age-related pathology including injury to the brain, retina, kidney, and liver, eventually causing disability and premature death in 100% of patients with these inherited mutations (Chauvin et al., 2024; Stam et al., 2016). The RVCL-associated C-terminal mutations therefore provide a window into the impact of DNA damage on aging separate from cytokine-mediated systemic inflammation.

## TREX1 dampens the DNA-sensing cGAS-STING pathway

**FIGURE 1:**
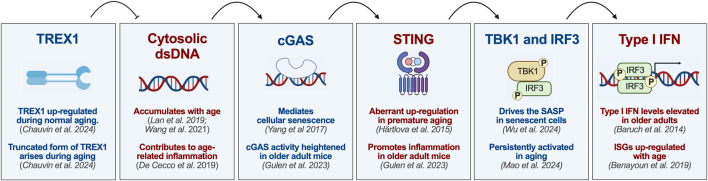
Role of the TREX1-cGAS-STING pathway in aging and cellular senescence. Cytosolic sensing of DNA is central to cellular senescence and the DNA damage theory of aging. TREX1 degrades cytosolic DNA, thereby preventing activation of the DNA sensor cGAS, which can mediate cellular senescence. Upon binding DNA, cGAS undergoes a conformational change that triggers the production of the second messenger cGAMP. After binding cGAMP, STING undergoes a conformation shift that promotes the recruitment and activation of the downstream signaling molecules TBK1 and IRF3, which are known to drive the senescence-associated secretory phenotype in senescent cells. Phosphorylated IRF3 translocates to the nucleus to facilitate the transcription of type I interferons (IFNs) and IFN-stimulated genes (ISGs), which are up-regulated with age. Figure created in BioRender. Miner, J. (2026) https://BioRender.com/vu8u3zx.

The cGAS-STING pathway is the key mediator of cellular responses to cytosolic dsDNA (Decout et al., 2021; Ablasser and Hur, 2020; Hopfner and Hornung, 2020; Chauvin et al., 2023). It serves to detect intracellular pathogens and instigate type I interferon and other inflammatory responses (Ablasser and Hur, 2020; Hopfner and Hornung, 2020; Chauvin et al., 2023). Upon recognition of cytosolic DNA, cGAS dimerizes and is activated to convert ATP and GTP to 2′,3′-cyclic -GMP–AMP (cGAMP) (Decout et al., 2021; Ablasser and Hur, 2020; Hopfner and Hornung, 2020; Chauvin et al., 2023). In turn, cGAMP binding to stimulator of interferon genes (STING) causes activation of TANK-binding kinase 1 (TBK1), phosphorylation of interferon regulatory factor 3 (IRF3), and transcription of type I interferon-associated genes (Figure 1) (Decout et al., 2021; Ablasser and Hur, 2020; Hopfner and Hornung, 2020; Chauvin et al., 2023) STING signaling also leads to NF-κB activation through a less well characterized mechanism (Decout et al., 2021; Hopfner and Hornung, 2020; Chauvin et al., 2023). However, the cGAS-STING pathway creates a significant risk of autoinflammation if excessively activated by self-DNA (Decout et al., 2021; Ablasser and Hur, 2020; Chauvin et al., 2023). Therefore, a number of mechanisms have evolved to protect the cell from improper cGAS-STING activation (Paul et al., 2021; Decout et al., 2021; Ablasser and Hur, 2020; Chauvin et al., 2023). These include a variety of nucleases, such as DNase II, SAMHD1, and TREX1, as well as compartmentalization of cellular DNA and post-translational modifications of cGAS and STING (Decout et al., 2021; Ablasser and Hur, 2020; Chauvin et al., 2023; Hong et al., 2022).

In the context of normal aging, cytosolic dsDNA accumulates, leading to activation of the cGAS-STING pathway, which promotes cellular senescence (Figure 1) (De Cecco et al., 2019; Wang et al., 2021; Yang et al., 2017; Wu et al., 2024; Mao et al., 2024) In premature aging, improper activation of the cGAS-STING pathway drives inflammation and the aging process. For example, Bloom syndrome results from the absence of Bloom syndrome protein, which causes accumulation of micronuclei, aberrant activation of cGAS-STING, and a syndrome associated with epigenetic features of aging and cancer predisposition (Gratia et al., 2019; Lee et al., 2023; Sugrañes et al., 2022). Likewise, cytoplasmic DNA accumulation in ataxia telangiectasia causes cGAS-STING activation and neurotoxicity (Lai et al., 2024; Härtlova et al., 2015). These observations in rare diseases support recent work revealing that the cGAS-STING pathway is responsible for inflammation and neurodegeneration in normal aging (Paul et al., 2021; Lan et al., 2019). In particular, Gulen and colleagues found that cGAS-STING activation is responsible for aberrant type I interferon signaling associated with aging-related responses in microglia (Gulen et al., 2023). Since cGAS and STING regulate cytokine responses during DNA damage, understanding the role of this pathway is crucial for defining the link between DNA damage and age-related inflammation.

TREX1 is an exonuclease that plays an important role in preventing autoinflammation due to cGAS-STING activation in response to aberrant self-DNA (Wang et al., 2022; Rice et al., 2015; Stetson et al., 2008). Physiologic TREX1 localizes to the cytoplasmic side of the endoplasmic reticulum and degrades cytoplasmic DNA (Wang et al., 2022; Rice et al., 2015). It is a homodimer of 314 amino acids, with the N-terminal domain responsible for catalytic activity and the C-terminal domain involved in localizing and anchoring to the endoplasmic reticulum (Wang et al., 2022; Rice et al., 2015; Zhou et al., 2022). This structure leads to a predictable connection between particular mutations and their phenotypic effects, with N-terminal mutants causing cytosolic DNA accumulation, cGAS-STING activation, and inflammation, while C-terminal mutants lead to mislocalization and DNA damage (Wang et al., 2022; Chauvin et al., 2024; Rice et al., 2015).

While TREX1 has not been comprehensively studied in normal aging, it is down-regulated in the peripheral blood of older humans, with more pronounced down-regulation observed in patients with autoimmune disease than healthy controls (Luo et al., 2023). Likewise, TREX1 is down-regulated in senescent fibroblasts, potentially contributing to the development of type I IFN responses and inducing the SASP (De Cecco et al., 2019; Takahashi et al., 2018). Down-regulation of TREX1 may explain, at least in part, the cGAS-STING pathway activation observed during senescence and aging (Gulen et al., 2023). However, it is unclear how this phenomenon crosstalks with other mechanisms of TREX1 regulation such as cytokine-induced up-regulation of TREX1 in animals (Chauvin et al., 2024; Serra et al., 2011). For example, TREX1 expression increases with age in the liver and brain of both mice and humans (Chauvin et al., 2024), as well as the peripheral blood of rats (Luo et al., 2023), suggesting that there may be context- or cell type-specific regulation of TREX1 levels during aging. Furthermore, additional research is needed to dissect the role of TREX1 in metabolism and other cellular processes central to normal aging beyond inflammation, DNA damage and senescence. However, research on TREX1 in normal aging is a new area of study, requiring further exploration to better define the impact of dynamic age-related changes in TREX1 levels across different tissues and cell types. Far more is known about disease-causing TREX1 mutants. Therefore, to begin to explore the role of TREX1 in aging, we next discuss disease-associated phenotypes resulting from TREX1 mutants and their relationship with the aging process.

## N-terminal mutations in TREX1 lead to inflammation with features of premature aging

**FIGURE 2:**
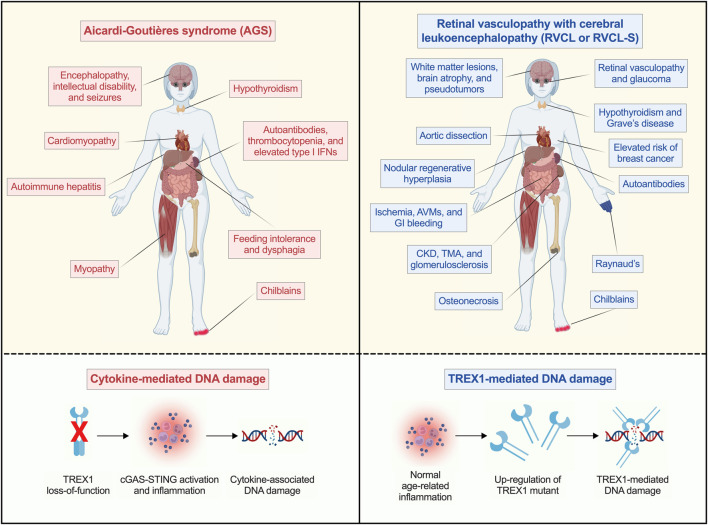
Distinct TREX1-related mechanisms cause DNA damage in the context of AGS and RVCL. Mutations in different regions of TREX1 cause completely distinct multi-organ diseases (AGS and RVCL) that both lead to universal disability and premature death. In AGS (top left), loss-of-function mutations in TREX1 cause inflammatory disease. In contrast, gain-of-function mutations in TREX1 cause RVCL (top right), but without systemic inflammation. In AGS, inflammation acts downstream of TREX1 mutations (bottom left), leading to cGAS-STING activation and inflammation. This can result in cytokine-associated DNA damage. In patients with RVCL, cytokines act upstream of TREX1 by up-regulating TREX1 protein expression with age (bottom right). In RVCL, age-related inflammation increases levels of a toxic mutant TREX1, which causes DNA damage. Figure created in BioRender. Miner, J. (2026) https://BioRender.com/xzlro22.

The most dramatic example of an N-terminal TREX1 mutation-associated phenotype is Aicardi-Goutières syndrome (AGS), a devastating type I interferonopathy that results in encephalopathy, intracranial calcifications, and severe developmental delay (Liu and Ying, 2023). Most patients with TREX1-associated AGS have very limited voluntary gross motor control and ability to communicate (Figure 2) (Adang et al., 2020) All genes associated with AGS have functions in nucleic acid sensing or nucleotide metabolism, suggesting that defects in these processes are responsible for the aberrant type I interferon activity in the disease (Liu and Ying, 2023).

Mutations in TREX1 cause some of the most severe disease phenotypes in AGS patients (Adang et al., 2020). Often, these are complete loss of function mutations, and most cause reduced catalytic activity (Hofer et al., 2024; Crow et al., 2015). Genetic deletion of TREX1 in mice also causes an autoinflammatory disease that is used as a model for AGS (Stetson et al., 2008). Notably, mouse models of AGS, resulting from biallelic TREX1-deficiency, are protected from lethality by genetic deletion of cGAS, STING, or the type I interferon receptor (IFNAR1) (Gall et al., 2012; Gao et al., 2015; Stetson et al., 2008; Gray et al., 2015). Intriguingly, Du and colleagues found that TREX1-deficient mouse models of AGS also display increased levels of DNA damage, and that this DNA damage and associated cellular senescence are reduced in the absence of cGAS (Du et al., 2023). We can thus construct a model of TREX1-associated AGS whereby deficiency in TREX1 leads to cytosolic DNA accumulation, triggering aberrant cGAS-STING activation and type I interferon activity, ultimately leading to irreversible organ injury and disease.

Initially, it may appear that AGS has little relation to aging research. AGS is not traditionally regarded as a premature aging disease, although the significant disability and increased risk of death in patients with TREX1 variants makes it difficult to confidently state whether premature aging of specific cell types and tissues occurs in this disease (Crow et al., 2015). Notably, TREX1-deficient mice experience premature lethality due to cardiac complications resulting from inflammatory myocarditis (Stetson et al., 2008; Morita et al., 2004). However, AGS’ utility in aging research might not come primarily from the phenotype observed in patients, but instead from defining the implications of DNA accumulation and cGAS-STING activation in the context of cellular injury. For example, Thomas and colleagues found that AGS model human neural stem cells lacking TREX1 accumulate Long Interspersed Element-1 (L1) retrotransposon DNA in the cytosol, leading to aberrant type I interferon activation and neuroinflammation (Thomas et al., 2017). This parallels more recent work by De Cecco and colleagues indicating that L1 accumulation is responsible for interferon activation and inflammation in senescent cells in normal aging (De Cecco et al., 2019).

One additional TREX1-associated type I interferonopathy is familial chilblain lupus (FCL) (Fiehn, 2017; Lee-Kirsch et al., 2007; Lee-Kirsch et al., 2006; Nohara et al., 2020). It is characterized by the presence of chilblains, inflammatory lesions primarily on the hands, feet, and ears that are often triggered by cold (Lee-Kirsch et al., 2007; Lee-Kirsch et al., 2006; Nohara et al., 2020). Some reports have also identified co-occurrence of cerebrovascular disease or other systemic manifestations in young adulthood in FCL patients (Nohara et al., 2020; Yamashiro et al., 2013; Günther et al., 2013). Peschke and colleagues also found upregulated type I interferon responses in blood of patients with TREX1-associated FCL (Peschke et al., 2014). Interestingly, some of the same point mutations in TREX1 have been observed to cause both FCL and AGS (Wang et al., 2022). However, mutations with milder effects on TREX1 exonuclease activity are more likely to cause FCL than those that ablate TREX1 activity (Wang et al., 2022; Lehtinen et al., 2008). Thus, FCL could be thought of as a cutaneous form of AGS, sharing a similar pathological mechanism, but with reduced accumulation of intracytoplasmic dsDNA, reduced type I interferon signaling, and a significantly milder clinical phenotype. Given the milder type I interferon response and longer lifespan, it may be fruitful to further explore mouse models of FCL in the context of cellular senescence and aging, especially for genetic variants resulting from mutations that can also cause AGS in humans.

## C-terminal mutations in TREX1 lead to DNA damage without systemic inflammation

C-terminal mutations in TREX1 cause retinal vasculopathy with cerebral leukoencephalopathy (RVCL or RVCL-S), an invariably fatal disease characterized by small vessel disease with retinopathy, brain lesions, liver disease, and renal injury, typically presenting in the fourth or fifth decades of life (Stam et al., 2016; Raynowska et al., 2018; Riley et al., 2017; Xie et al., 2021). Inheritance is autosomal dominant, and the disease has 100% penetrance in mutation carriers (Richards et al., 2007). Death often occurs within 5–15 years of initial presentation (Stam et al., 2016; Xie et al., 2021; Richards et al., 2007). Patients also display other consequences of small vessel disease, including liver and kidney dysfunction (Figure 2) (Stam et al., 2016; Raynowska et al., 2018; Xie et al., 2021; Richards et al., 2007) While RVCL is often misdiagnosed as systemic lupus erythematosus (SLE), the distinct pathologic mechanism in RVCL clearly distinguishes it from SLE.

All RVCL-associated genetic variants truncate TREX1 at its C-terminus, which is responsible for localization of the protein to the cytosolic side of the endoplasmic reticulum (Richards et al., 2007). While some previously suggested that RVCL-mutant TREX1 is associated with interferonopathy, more recent studies of large patient cohorts and animal models clearly indicate a lack of systemic inflammation in RVCL (Chauvin et al., 2024; Rodero et al., 2017). More recent work revealed that RVCL is driven by mislocalization of TREX1 to the nucleus, leading to DNA damage and cellular senescence (Chauvin et al., 2024; McGlasson et al., 2025). In response to cytokines such as type I and type II IFN, the RVCL-causing form of TREX1 is highly up-regulated, resulting in high levels of DNA damage (Chauvin et al., 2024). This implicates a model in which cytokines act upstream of TREX1 to cause genome instability and pathology. The molecular mechanisms underlying DNA damage in RVCL remain an active area of study. An enzyme-dead TREX1 mutant does not cause DNA damage, suggesting that the exonuclease activity of TREX1 directly acts on genomic DNA (Chauvin et al., 2024). However, an alternative model is that TREX1 indirectly disrupts DNA damage repair. Indeed, TREX1 associates with PARP1 and has been implicated in DNA repair mechanisms (Yang et al., 2007; Christmann et al., 2010; Miyazaki et al., 2014), although this process remains incompletely understood. In further support of RVCL as a DNA damage syndrome, this disease is also associated with disrupted homology-directed repair and increased risk of early-onset breast cancer, similar to patients with genetic variants in *BRCA1* and *BRCA2* (Chauvin et al., 2024). Furthermore, patients with RVCL may have particularly adverse reactions to DNA-damaging chemotherapeutic agents (Chauvin et al., 2024).

Combined, these data allow us to propose a model for RVCL, where cells harboring the RVCL-associated TREX1 mutants do not themselves have increased inflammation. Instead, cells expressing the RVCL-causing mutant are less tolerant of inflammatory signals and DNA damage. Given that sterile inflammation and accumulation of DNA damage are both hallmarks of aging (Franceschi et al., 2000; Baruch et al., 2014; Benayoun et al., 2019), this can be interpreted as increased susceptibility to cellular processes that drive aging. Thought of in this way, the RVCL clinical phenotype of small vessel disease, more commonly seen in older patients (Cannistraro et al., 2019), can potentially be interpreted as a kind of premature aging. Indeed, the pathological findings in RVCL and other hereditary small vessel diseases are qualitatively similar to those of sporadic small vessel disease (Craggs et al., 2014). Because of these similarities, RVCL has been proposed as a model for sporadic small vessel disease (Wilms et al., 2022). The neuropathological findings observed in RVCL also mimic radiation necrosis, supporting a role for DNA damage in RVCL pathology (Kolar et al., 2014). Interestingly, these mechanisms may also intersect with those of laminopathy-induced DNA damage. In progeroid laminopathies, rupture of the fragile nuclear membrane leads to DNA damage and premature aging (Marcelot et al., 2021; Shin and Worman, 2022). Intriguingly, Nader and colleagues have observed DNA damage after nuclear envelope rupture in the context of cancer, and they proved this to be TREX1-dependent even in the context of full-length TREX1, which can translocate to the nucleus under certain conditions (Miyazaki et al., 2014; Nader et al., 2021). Thus, RVCL may serve as a model system to assess the effects of nuclear TREX1 on small vessel disease, cancer, age-related inflammation, and cellular senescence.

## TREX1-associated diseases underscore distinct mechanisms associated with aging

One frequent misdiagnosis in RVCL patients is multiple sclerosis (MS), and especially tumefactive MS (Raynowska et al., 2018; Hardy et al., 2017). This is based not only on the presence of similar focal neurologic findings, but also that the observed brain lesions are edematous, and that they wax and wane in severity, classically more consistent with an immune pathology than with ischemia (Hardy et al., 2017). Intriguingly, vascular dysfunction, especially age-related small vessel disease, has been proposed as a key contributor to or even driver of MS pathology (Spencer et al., 2018; Geraldes et al., 2017). As RVCL does not cause the immune-mediated demyelination that is a hallmark of MS, it presents a fascinating opportunity to evaluate the effects of small vessel disease in isolation from immune dysfunction or systemic inflammation seen in normal aging.

In normal aging, it is difficult to distinguish between inflammation that is downstream of DNA damage or telomere dysfunction and inflammation as a driver of other pathological mechanisms. By contrast, in AGS and FCL the sterile inflammation downstream of cGAS-STING activation drives the observed phenotypes. Thus, AGS, FCL, and their mouse models are useful platforms for exploring the contributions of inflammation to the aging process, especially in cell types known to undergo robust activation of the cGAS-STING pathway.

## Conclusion

TREX1-associated diseases engage several fundamental mechanisms that drive the normal aging process, thereby creating disease and cellular phenotypes that recapitulate features of premature aging. Crucially, however, distinct disease-causing mutations at the N- and C-termini of TREX1 elucidate and isolate two separate mechanisms that are nigh-inseparable during normal aging: inflammation and DNA damage. In the case of C-terminal TREX1 mutations, RVCL specifically implicates DNA damage and associated vasculopathy without any detectable systemic inflammatory effects. By contrast, N-terminal TREX1 mutations in AGS and FCL trigger immunological phenotypes associated with robust systemic inflammation, which may secondarily cause genome instability. Taken together, this suggests that experimental manipulation of TREX1 can play an important role in aging research.

We propose a model for conceiving of TREX1-associated diseases in terms of both regulating inflammation, as well as determining the response to normal age-related inflammation. On one end, AGS results in increased inflammation. On the other, RVCL is characterized by decreased *tolerance of* inflammation, leading to DNA damage (Figure 2). Thus, AGS serves as a useful model of inflammatory responses linked to physiologic aging as well as premature aging. RVCL, on the other hand, may be well-suited as a model for physiologic or premature aging without systemic inflammation, allowing for the utilization of models that undergo aging-associated DNA damage in the absence of hypercytokinemia. Future research may further develop these models as platforms for fundamental discovery in aging research.
